# Identification of neutrophil extracellular trap-driven gastric cancer heterogeneity and C5AR1 as a therapeutic target: Identification of NET-driven GC heterogeneity and C5AR1 as a therapeutic target

**DOI:** 10.3724/abbs.2023290

**Published:** 2024-03-01

**Authors:** Jing Zhao, Xiangyu Li, Liming Li, Beibei Chen, Weifeng Xu, Yunduan He, Xiaobing Chen

**Affiliations:** 1 Department of Medical Oncology Affiliated Cancer Hospital of Zhengzhou University Henan Cancer Hospital Zhengzhou 450003 China; 2 Department of Radiation Oncology Affiliated Cancer Hospital of Zhengzhou University Henan Cancer Hospital Zhengzhou 450003 China; 3 Department of Radiology the First Affiliated Hospital of Zhengzhou University Zhengzhou 450052 China; 4 State Key Laboratory of Esophageal Cancer Prevention and Treatment Zhengzhou University Zhengzhou 450052 China

**Keywords:** neutrophil extracellular trap, gastric cancer, heterogeneity, personalized therapy, C5AR1, treatment target

## Abstract

Neutrophil extracellular traps (NETs) are implicated in gastric cancer (GC) growth, metastatic dissemination, cancer-associated thrombosis, *etc*. This work is conducted to elucidate the heterogeneity of NETs in GC. The transcriptome heterogeneity of NETs is investigated in TCGA-STAD via a consensus clustering algorithm, with subsequent external verification in the GSE88433 and GSE88437 cohorts. Clinical and molecular traits, the immune microenvironment, and drug response are characterized in the identified NET-based clusters. Based upon the feature genes of NETs, a classifier is built for estimating NET-based clusters via machine learning. Multiple experiments are utilized to verify the expressions and implications of the feature genes in GC. A novel NET-based classification system is proposed for reflecting the heterogeneity of NETs in GC. Two NET-based clusters have unique and heterogeneous clinical and molecular features, immune microenvironments, and responses to targeted therapy and immunotherapy. A logistic regression model reliably differentiates the NET-based clusters. The feature genes *C5AR1*, *CSF1R*, *CSF2RB*, *CYBB*, *HCK*, *ITGB2*, *LILRB2*, *MNDA*, *MPEG1*, *PLEK*, *SRGN*, and *STAB1* are proven to be aberrantly expressed in GC cells. Specific knockdown of *C5AR1* effectively hinders GC cell growth and elicits intracellular ROS accumulation. In addition, its suppression suppresses the aggressiveness and EMT phenotype of GC cells. In all, NETs are the main contributors to intratumoral heterogeneity and differential drug sensitivity in GC, and C5AR1 has been shown to trigger GC growth and metastatic spread. These findings collectively provide a theoretical basis for the use of anti-NETs in GC treatment.

## Introduction

Gastric cancer (GC) represents a global health-care challenge [1]. The therapeutic landscape of GC has markedly evolved. Although the effectiveness of chemotherapy and surgical removal has improved, patient prognosis remains unsatisfactory [2]. Chemicalotherapeutic agents with low toxicity, along with molecular-driven targeted treatment, are valuable in sequential therapeutic regimens for the optimization of patient survival. In advanced disease, only trastuzumab and immune checkpoint blockade (ICB), *e*. *g*., nivolumab and pembrolizumab in combination with chemotherapy, have shown durable and superior effects in treating HER2-positive and PD-L1-positive patients, respectively [ 3‒ 5]. Biomarkers currently utilized for therapeutic choices include HER2 overexpression and amplification, combined positive PD-L1 score, and microsatellite instability. [6]. Intra- and intertumor heterogeneity are notable features of GC and are partially responsible for unfavorable survival [ 7, 8]. Nonetheless, only histological classification is not sufficient to potently stratify patients for personalized therapy or to prolong survival time. Large-scale molecular characterization of GC patients is important for identifying candidate treatment options [9].

Neutrophils are the dominating leukocytes in peripheral blood and are the initial defense against invading pathogens [ 10‒ 12]. Neutrophil extracellular traps (NETs) are network structures comprising decondensed DNA strands coated with granule proteins that were first discovered in 1996 [13], and these NETs were subsequently named NETosis [ 14, 15]. Based upon mounting evidence, circulating NET levels are aberrantly increased in GC patients, and NETs have indispensable implications for GC growth [16], the metastatic cascade [ 17‒ 19] and cancer-associated thrombosis [20]. However, additional studies on NET formation and compounds that hinder or destroy NETs are needed, providing a theoretical basis for the use of NETs in GC treatment.

In the present study, a novel NET-based molecular classification system for GC was proposed, revealing the heterogeneity of NET formation. In addition, a classifier was built for the differentiation of NET-based clusters. Among the feature genes in the classifier, C5AR1 was experimentally proven to mediate GC growth and metastatic spread, indicating the potential of C5AR1 as a treatment target in GC.

## Materials and Methods

### Human GC data acquisition

Using the TCGA database ( https://portal.gdc.cancer.gov/), GC RNA sequencing and clinical data from the TCGA-STAD cohort were curated, and the GC expression matrix and clinical traits of the GSE88433 and GSE88437 cohorts were subsequently acquired from the GEO database ( https://www.ncbi.nlm.nih.gov/gds/) [21]. Utilizing edgeR [22], the raw data were normalized.

### Consensus clustering analysis

Thirty-five NET-associated genes were identified from prior research [23] and are summarized in Supplementary Table S1. Based upon their expression profiles, consensus clustering analysis was performed across TCGA-STAD samples utilizing ConsensusClusterPlus [24]. The K values of the clusters were set as 2 to 5, with subsequent optimal selection based upon the consensus matrix. Such clustering was proven in GSE88433 and GSE88437. Differential overall survival (OS) and disease-free survival (DFS) outcomes were evaluated in distinct clusters.

### Somatic variant analysis

The mutation annotation format of the TCGA-STAD cohort was adopted from the TCGA-STAD cohort [25]. The mutation frequency of genes was estimated and compared between clusters by using Fisher’s exact test.

### Characterization of cell compositions

CIBERSORT, a deconvolution approach [26], was adopted for estimating immune compositions within GC tumors. The fractions of stromal and immune cell admixture were inferred via the ESTIMATE method [27].

### Functional enrichment analysis

GSEA software was used for enrichment analyses of GO and KEGG terms [28]. In addition, enrichment levels of classical tumorigenic signaling pathways were scored via the GSVA package [29].

### Treatment response estimation

Based on the GDSC2 database [30], the IC _50_ of small-molecule agents was inferred via oncoPredict [31]. The ICB response was estimated in accordance with the tumor mutational burden (TMB), T-cell-inflamed score [32], and tumor immune dysfunction and exclusion (TIDE) score [33].

### Development of a classifier

First, DEGs with a |log2 fold change|>1 and q<0.05 were selected by comparing two NET-based clusters. Gene expression levels were utilized as the prediction value, and the area under the curve (AUC) was estimated. In accordance with the AUC values, the top twelve genes were chosen as feature genes. Through the Python machine learning framework sklearn, linear function support vector machine (linearSVM), radial basis function SVM (RBFsvm), Gaussian naive Bayes (GaussianNB), and logistic regression (logistic) models were trained. Receiver operating characteristic (ROC) analysis was also conducted to evaluate the prediction efficacy of the pROC package.

### Patients and clinical specimens

In total, 106 GC patients were recruited from Henan Cancer Hospital. Tumor tissue specimens were collected following institutional protocols. Prognostic information was also obtained. Each participant provided written informed consent. This study was approved by the Ethics Committee of Henan Cancer Hospital (No. 2020012).

### Cell culture and transfection

Normal human gastric mucosal epithelial cells (GES-1) and GC cell lines (AGS, SGC7901 and MKN28) were purchased from the Typical Culture Preservation Committee Cell Bank of the Chinese Academy of Sciences (Shanghai, China). All the cells were cultivated in RPMI-1640 medium (12633012; Gibco, Carlsbad, USA) supplemented with 10% FBS (SH30396.03; HyClone, Carlsbad, USA). The cells were placed in a 5% CO _2_ cell incubator (SW-CJ-1FD; Thermo Fisher Scientific, Waltham, USA) at 37°C. The medium was changed every 1‒2 days.

Three siRNAs against C5AR1 (si-C5AR1) and a negative control (si-NC) ( Table 1) purchase from Sangon Biotech (Shanghai, China) were transfected into GC cells via Lipofectamine 2000 (11668030; Invitrogen, Carlsbad, USA) based on the manufacturer’s instructions. The transfection efficiency was verified via western blot analysis. The optimal si-C5AR1 was subsequently chosen.

**Table:** **Table 1** Sequences of primers and siRNAs used in this study

Gene	Sequence (5′→3′)
*C5AR1*	F: TCCTTCAATTATACCACCCCTGA
	R: ACGCAGCGTGTTAGAAGTTTTAT
*CSF1R*	F: GGGAATCCCAGTGATAGAGCC
	R: TTGGAAGGTAGCGTTGTTGGT
*CSF2RB*	F: AGCGGCTTCAGGACTCTTG
	R: CTGGGCATGAGGTGCTCTG
*CYBB*	F: ACCGGGTTTATGATATTCCACCT
	R: GATTTCGACAGACTGGCAAGA
*HCK*	F: CCCTGTATGATTACGAGGCCA
	R: CACTCCCCGGATTCCTCTAGG
*ITGB2*	F: TGCGTCCTCTCTCAGGAGTG
	R: GGTCCATGATGTCGTCAGCC
*LILRB2*	F: GCATCTTGGATTACACGGATACG
	R: CTGACAGCCATATCGCCCTG
*MNDA*	F: AACTGACATCGGAAGCAAGAG
	R: CCTGATTCGGAGTAAACGAAGTG
*MPEG1*	F: CGGCAGCATGGGCTAAATCA
	R: TGTCCACATTCCGCAGATTGT
*PLEK*	F: AAGAAGGGGAGCGTGTTCAAT
	R: TCAGCGGGATCATTCCTTTGG
*SRGN*	F: AGGTTATCCTACGCGGAGAG
	R: GTCTTTGGAAAAAGGTCAGTCCT
*STAB1*	F: AACCACGTTTGTCACTCATGT
	R: CGGCAGTCCTGGGTTATCTG
*GAPDH*	F: ACAACTTTGGTATCGTGGAAGG
	R: GCCATCACGCCACAGTTTC
si-NC	UUCUCCGAACGUGUCACGUTT
si-C5AR1 (1)	GCUUUCUGCUGGUGUUUAAAC
si-C5AR1 (2)	GGUCCACCAAGACACUCAAGG
si-C5AR1 (3)	GCUCUUGUAAGUGAGUUAAUU

### RT-qPCR analysis

Cells or tissues were lysed with Trizol (H10318; TransGen, Beijing, China), after which RNA was extracted. The RNA concentration was measured via a Nanodrop 2000 spectrophotometer (Thermo Fisher Scientific). Before quantification, the extracted RNA was stored at ‒80°C. Complementary DNA was subsequently synthesized. A real-time PCR kit (AQ131-01; TransGen) and SYBR Green Master Mix (Rainbio, Shanghai, China) were used to perform RT-qPCR. Table 1 summarizes the primer sequences utilized for RT-qPCR. The 2 ^‒ΔΔCt^ method was used to quantify mRNA expression.

### Western blot analysis

Cells were lysed in RIPA cell lysis buffer supplemented with a protease inhibitor mixture, followed by centrifugation and supernatant collection. Following SDS-PAGE, the proteins were transferred onto PVDF membranes (Millipore, Billerica, USA) which were subsequently blocked. Next, the membranes were incubated with primary anti-C5AR1 (1:500; MA5-16937; Invitrogen) or anti-GAPDH (1:3000; AF7021; Affinity, Changzhou, China) antibodies at 4°C overnight, followed by incubation with HRP-labelled secondary antibody (1:3000; S0001; Affinity) for 1 h at room temperature. The protein bands were developed using an Enhanced chemiluminescence (ECL) luminous substrate (P90719; Millipore) and photographed with a chemiluminescence imaging system (ChemiScop Series 3600; Enco Biology, Shenzhen, China).

### Cell viability test

Cell viability was determined using the CCK8 detection kit (C0038; Beyotime, Shanghai, China) following the manufacturer’s instructions. Briefly, cells were seeded in 96-well plates. After culture and treatment, 10 μL of CCK8 reagent was added to each well and incubated for 1 h. Then, the absorbance at 450 nm was measured utilizing a microplate reader (Multiskan MK3; Thermo Fisher Scientific).

### Reactive oxygen species (ROS) measurement

ROS levels were measured using a DCFH-DA kit (E-BC-K138-F; Elabscience, Wuhan, China) according to the manufacturer’s instructions. A single-cell suspension was added to the working solution. The cells were incubated at 37°C for 1 h in the dark and centrifuged at 1000 *g* for 10 min. The gathered cell precipitate was resuspended in the working fluid. Finally, ROS levels were tested with a flow cytometer (FACSCalibur; BD, Franklin Lakes, USA).

### Wound healing assay

Approximately 5×10 ^5^ cells were added to each well of a 6-well plate which was marked on the back. The next day, scratches were made vertically to the horizontal line on the back. Following removal of the scratched cells, serum-free medium was added. At 0 and 48 h, the cells were photographed.

### Immunofluorescence microscopy

Following fixation with 4% polyformaldehyde, the cells were permeated with PBS containing 0.1% Triton X-100 (Sigma-Aldrich, St Louis, USA), and the cells were subsequently blocked with 5% BSA sealing solution. Next, the cells were incubated with anti-E-cadherin (1:500; AF0131; Affinity) or anti-Vimentin (1:500; AF7013; Affinity) antibody for 1 h at room temperature, and then with a secondary antibody (1:500; ab6717 or ab150083; Abcam) for 1 h in the dark. The cell nuclei were then stained with DAPI (C1002; Beyotime) for 5 min. Finally, images were captured with a fluorescence microscope (DMI3000B; Leica, Wetzlar, Germany).

### Statistical analysis

All the analyses were conducted with the R platform (version 3.6.1) or GraphPad Prism software (version 9.0.1). Pearson’s or Spearman’s test was adopted for correlation analysis. Differences between two groups were estimated with Student’s *t* test, the Wilcoxon test, or the chi-square test, with one-way ANOVA for comparisons among more than 3 groups. *P*<0.05 was considered statistically significant.

## Results

### Stratification of GC patients into two NET-based clusters

**Figure 1:**
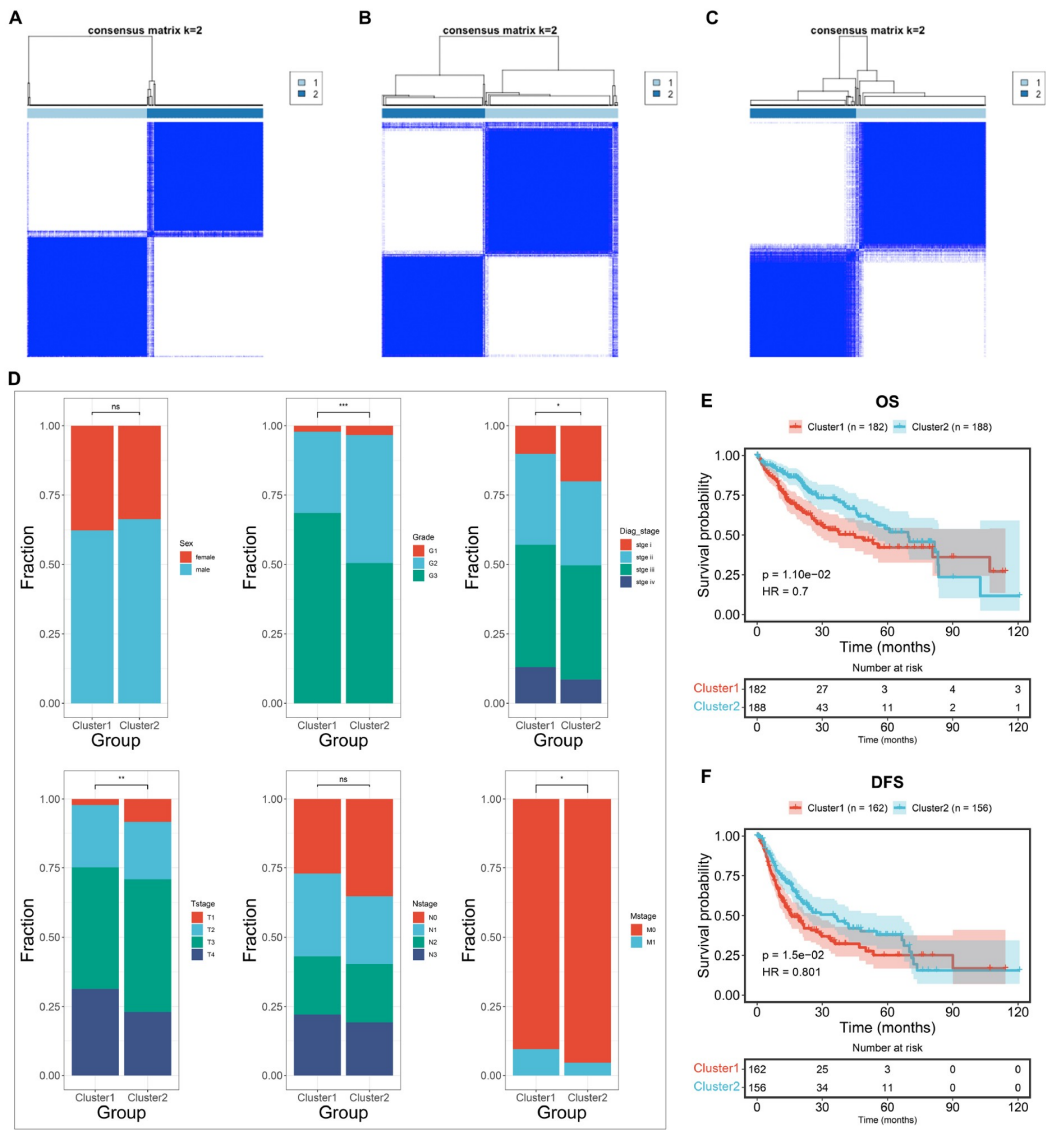
Figure 1 Stratification of GC patients into two NET-based clusters (A) Consensus matrix heatmap (k=2) representing the possibility of two patients belonging to the same cluster across TCGA-STAD samples based upon NET-associated genes. The value ranges from 0 to 1, and the color ranges from white to dark blue. (B,C) Verification of the NET-based consensus clustering analysis in GSE88433 and GSE88437. (D) Heterogeneous clinical traits of two NET-based clusters in the TCGA-STAD cohort. ns, not significant; *P<0.05, **P<0.01, and ***P<0.001. (E,F) Distinct OS and DFS probabilities between the two clusters.

To characterize the heterogeneity of NETs across GCs, we conducted consensus clustering analysis of TCGA-STAD samples based on the expressions of known NET-associated genes. After obtaining a consensus matrix, the TCGA-STAD samples were clearly stratified into two clusters: cluster 1 and cluster 2 ( Figure 1A). The consistency and reliability of this molecular classification system were externally proven in the GSE88433 and GSE88437 cohorts ( Figure 1B,C). Additionally, heterogeneous clinical traits were investigated between the clusters. In particular, cluster 1 had more advanced tumor grade, stage, and TNM stage ( Figure 1D). Cluster 1 patients also presented shorter OS and DFS time than cluster 2 patients ( Figure 1E,F). Taken together, these findings suggested that the two NET-based clusters are clinically heterogeneous in GC.

### Differences in heterogeneous somatic variants and immune compositions between NET-based clusters

**Figure 2:**
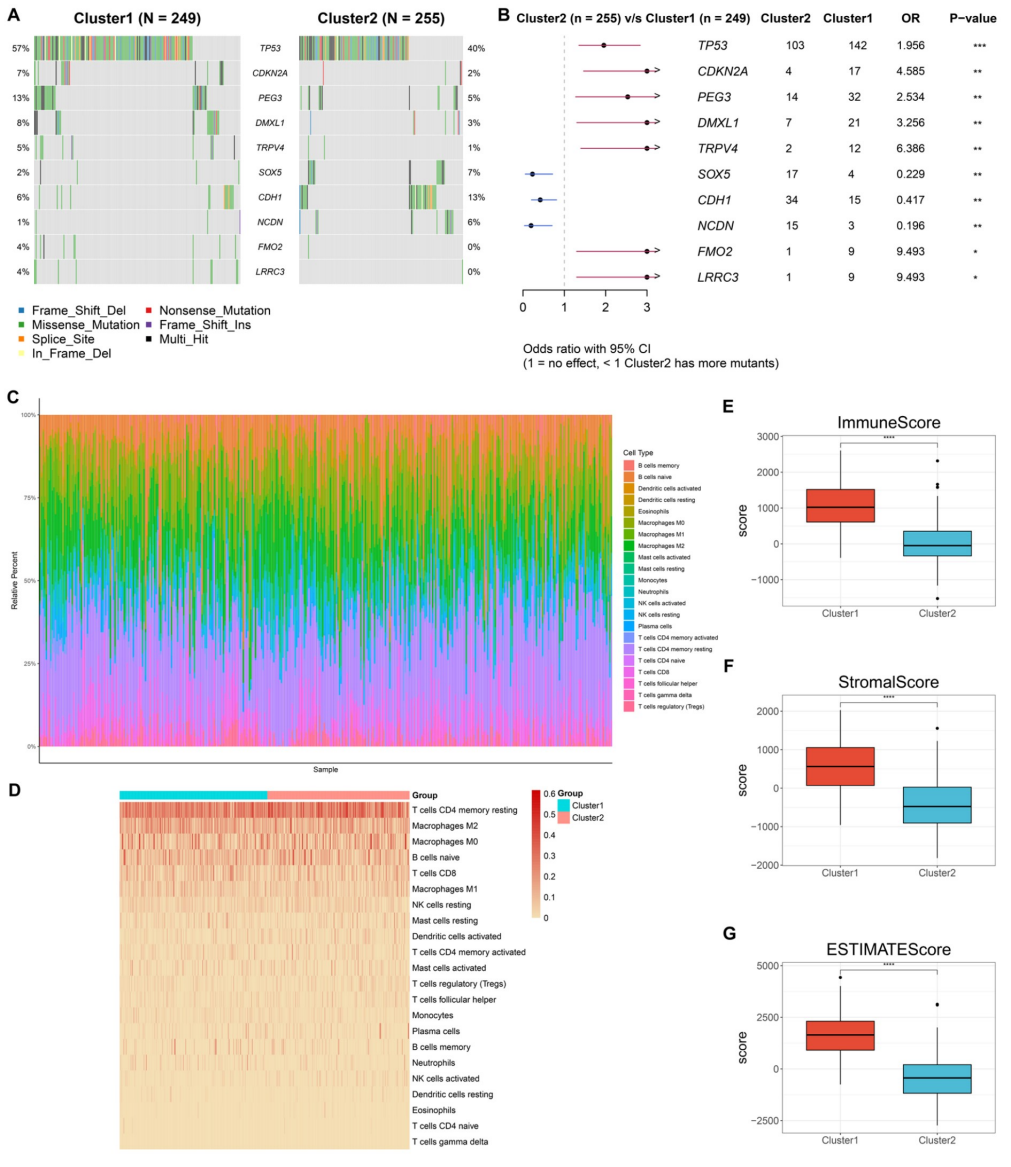
Figure 2 Differences in heterogeneous somatic variants and immune compositions between the two NET-based clusters (A) Oncoplots illustrating the somatic landscape of two NET-based clusters. Genes are ranked based upon mutational frequency. (B) Forest plot of genes differentially mutated between clusters. (C) Immune composition landscape across TCGA-STAD specimens. (D) Visualization of the fractions of immune compositions in TCGA-STAD patients stratified by NET-based classification. (E–G) Differential immune, stromal or ESTIMATE scores between NET-based clusters. *P<0.05, **P<0.01, ***P<0.001 and ****P<0.0001.

Analysis of somatic variants revealed that TP53, CDKN2A, PEG3, DMXL1, TRPV4, FMO2, and LRRC3 had more frequent mutations in cluster 1 than in cluster 2, with more frequent mutations in SOX5, CDH1, and NCDN occurring in cluster 2 ( Figure 2A,B), revealing the unique molecular mutation traits in each cluster. In addition, most immune components appeared to be more abundant in cluster 1 than in the other clusters ( Figure 2C,D). Higher immune, stromal and ESTIMATE scores were found in cluster 1 ( Figure 2E‒G), further reflecting the richer stromal and immune cell admixtures in this cluster.

### Heterogeneous molecular mechanisms between NET-based clusters

**Figure 3:**
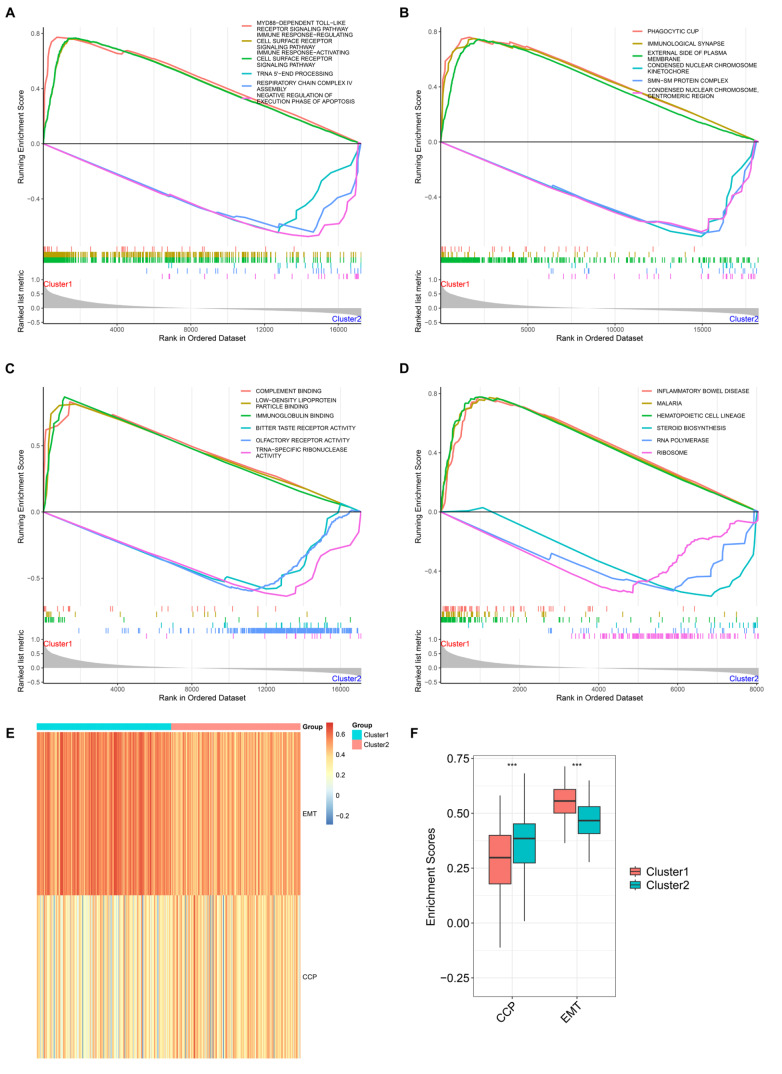
Figure 3 Heterogeneous molecular mechanisms in the two NET-based clusters (A‒D) GSEA for the enrichment analysis of biological processes, cellular components, molecular functions, and KEGG pathways in the two clusters. (E,F) Characterization and comparison of the enrichment scores of EMT and CCP in the two clusters. ***P<0.001.

The molecular mechanisms underlying the NET-based classification were subsequently explored. Consequently, immunity-associated mechanisms were notably activated in cluster 1, with remarkable activation of metabolic processes in cluster 2 ( Figure 3A‒D). In addition, epithelial–mesenchymal transition (EMT) activity was greater in cluster 1, and cell cycle progression (CCP) activity was greater in cluster 2 ( Figure 3E,F). The above findings reflected the heterogeneity in the molecular mechanisms of the two NET-based clusters.

### Heterogeneity in treatment responses between NET-based clusters

Next, we investigated whether two NET-based clusters elicit diverse therapeutic responses. Among the small-molecule compounds, cluster 2 was more sensitive to BI-2536, with greater sensitivity to KU-55933, ribociclib, AZD8186, AZD8055, NU7441, SB216763, BMS-754807, AZ960, entospletinib, ZM447439, CZC24832, AMG-319, GSK269962A, WZ4003, JQ1, RVX-208, and PRT062607 in cluster 1 ( Supplementary Figure S1A). Three ICB predictors, TMB, T-cell-inflamed status and TIDE score, were adopted for inferring the ICB response. Cluster 2 tumors exhibited greater TMB than cluster 1 tumors, but the difference was not significant ( Supplementary Figure S1B,C). Moreover, higher T-cell-inflamed and TIDE scores were detected in cluster 1 than in another cluster ( Supplementary Figure S1D,E). Hence, the two NET-based clusters exhibited diverse responses to targeted therapy and ICB.

### Establishment of a stable and reliable classifier for differentiating NET-based clusters

**Figure 4:**
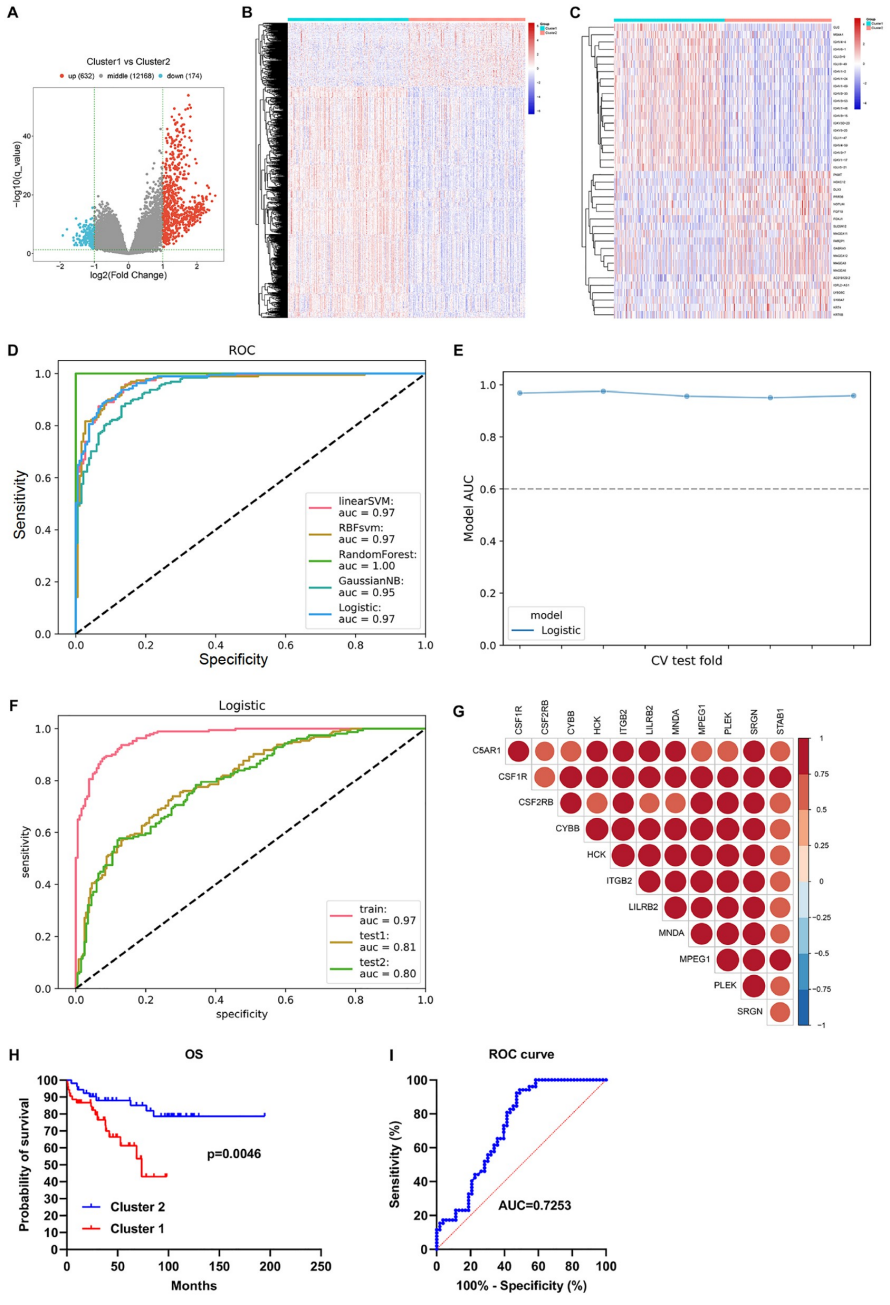
Figure 4 Definition of a stable and reliable classifier for differentiating between two NET-based clusters (A,B) Selection of genes with differential expression between clusters. The cut-off was |log2-(fold change)|>1 and q<0.05. (C) The first twenty genes in each cluster. (D) ROC analysis of the established linear SVM, RBFsvm, GaussianNB, and logistic models in the TCGA-STAD cohort. (E) Fivefold cross-validation curves of the logistic model. (F) External verification of the logistic model in the GSE88433 and GSE88437 cohorts. (G) Correlation analysis of the twelve feature genes. (H) OS probability of patients in clusters 1 and 2 in a real-world GC patient cohort (n=106). (I) ROC curves for the classification of OS in the cohort.

**Figure 5:**
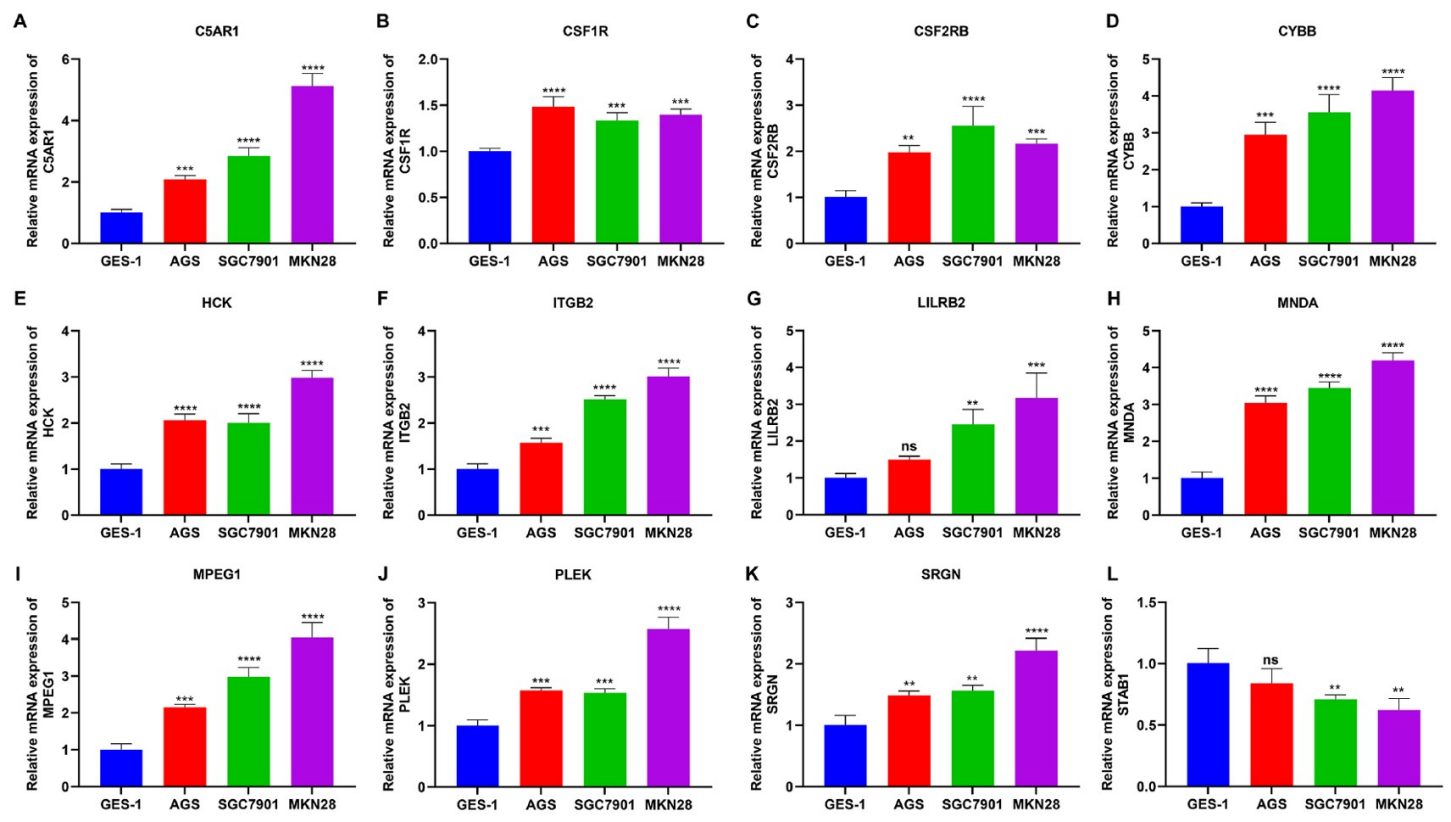
Figure 5 Experimental verification of feature genes in GC and normal gastric epithelial cells (A‒L) RT-qPCR was used to measure the expressions of C5AR1, CSF1R, CSF2RB, CYBB, HCK, ITGB2, LILRB2, MNDA, MPEG1, PLEK, and SRGN in GES-1, AGS, SGC7901 and MKN28 cells. ns, not significant; **P<0.01, ***P<0.001 and ****P<0.0001.

To differentiate the two NET-based clusters, we first selected genes differentially expressed between the clusters ( Figure 4A‒C). With an AUC>0.9, twelve feature genes of the NET-based classification ( *C5AR1*, *CSF1R*, *CSF2RB*, C *YBB*, *HCK*, *ITGB2*, *LILRB2*, *MNDA*, *MPEG1*, *PLEK*, *SRGN*, and *STAB1*) were chosen for building five classifiers (linearSVM, RBFsvm, GaussianNB, and logistic models). All the feature genes had higher expression in cluster 1 than in cluster 2 ( Supplementary Figure S2). To verify the efficacy of these classifiers in differentiating the two NET-based clusters, receiver operating characteristic (ROC) analysis was conducted. The AUC of the random forest model was 1 for TCGA-STAD cohort, indicating possible data overfitting ( Figure 4D). The AUC values of the logistic model were relatively higher, and were 0.970, 0.806 and 0.796 in the TCGA-STAD, GSE88433 and GSE88437, respectively ( Figure 4E,F and Table 2). Thus, the logistic model was eventually utilized for differentiating the two NET-based clusters. Table 3 shows the weight of each feature gene in the logistic model. These feature genes were found to be strongly related to each other ( Figure 4G). To further validate the performance of the classifier in determining GC prognosis, a real-world cohort of GC patients ( *n*=106) was included in our study. Patients were classified into cluster 1 and cluster 2 according to the weight and expression of feature genes. The results showed that cluster 1 patients had shorter OS than cluster 2 patients ( Figure 4H), and the AUC was 0.7253 ( Figure 4I), further confirming the stability and reliability of the classifier. In addition, C5AR1, CSF1R, CSF2RB, CYBB, HCK, ITGB2, LILRB2, MNDA, MPEG1, PLEK, and SRGN exhibited notable upregulation in GC cells (AGS, SGC7901 and MKN28) compared with those in GES-1 cells, with remarkable downregulation of STAB1 expression ( Figure 5).

**Table:** **Table 2** Analysis of the efficacy of five machine learning classifiers in differentiating two NET-based clusters

Dataset	Classifier	AUC	95%CI	*P*	Sensitivity	Specificity
TCGA-STAD	linearSVM	0.969	0.955‒0.983	0	0.958	0.859
	RBFsvm	0.966	0.949‒0.983	0	0.948	0.870
	RandomForest	1	1‒1	0	1	1
	GaussianNB	0.949	0.929‒0.968	0	0.874	0.870
	Logistic	0.970	0.956‒0.984	0	0.885	0.924
GSE88433	linearSVM	0.798	0.757‒0.839	2.09×10 ^‒46^	0.595	0.845
	RBFsvm	0.785	0.742‒0.829	8.88×10 ^‒38^	0.713	0.769
	RandomForest	0.818	0.779‒0.857	2.22×10 ^‒57^	0.779	0.727
	GaussianNB	0.838	0.801‒0.874	1.76×10 ^‒73^	0.749	0.773
	Logistic	0.806	0.766‒0.846	1.66×10 ^‒50^	0.738	0.723
GSE88437	linearSVM	0.791	0.745‒0.837	1.44×10 ^‒35^	0.545	0.891
	RBFsvm	0.774	0.726‒0.823	3.39×10 ^‒28^	0.724	0.736
	RandomForest	0.803	0.758‒0.847	1.98×10 ^‒40^	0.667	0.776
	GaussianNB	0.829	0.788‒0.871	9.06×10 ^‒55^	0.712	0.811
	Logistic	0.796	0.751‒0.842	3.42×10 ^‒37^	0.577	0.876

**Table:** **Table 3** The weight of each feature gene in the logistic model

Feature	Weight
C5AR1	1.301465
CSF1R	0.798827
CSF2RB	1.691757
CYBB	‒0.93195
HCK	0.176422
ITGB2	0.370265
LILRB2	0.393421
MNDA	0.055314
MPEG1	‒0.74223
PLEK	0.56748
SRGN	0.51999
STAB1	0.911245

### Knockdown of the feature gene *C5AR1* prevents GC cell growth

**Figure 6:**
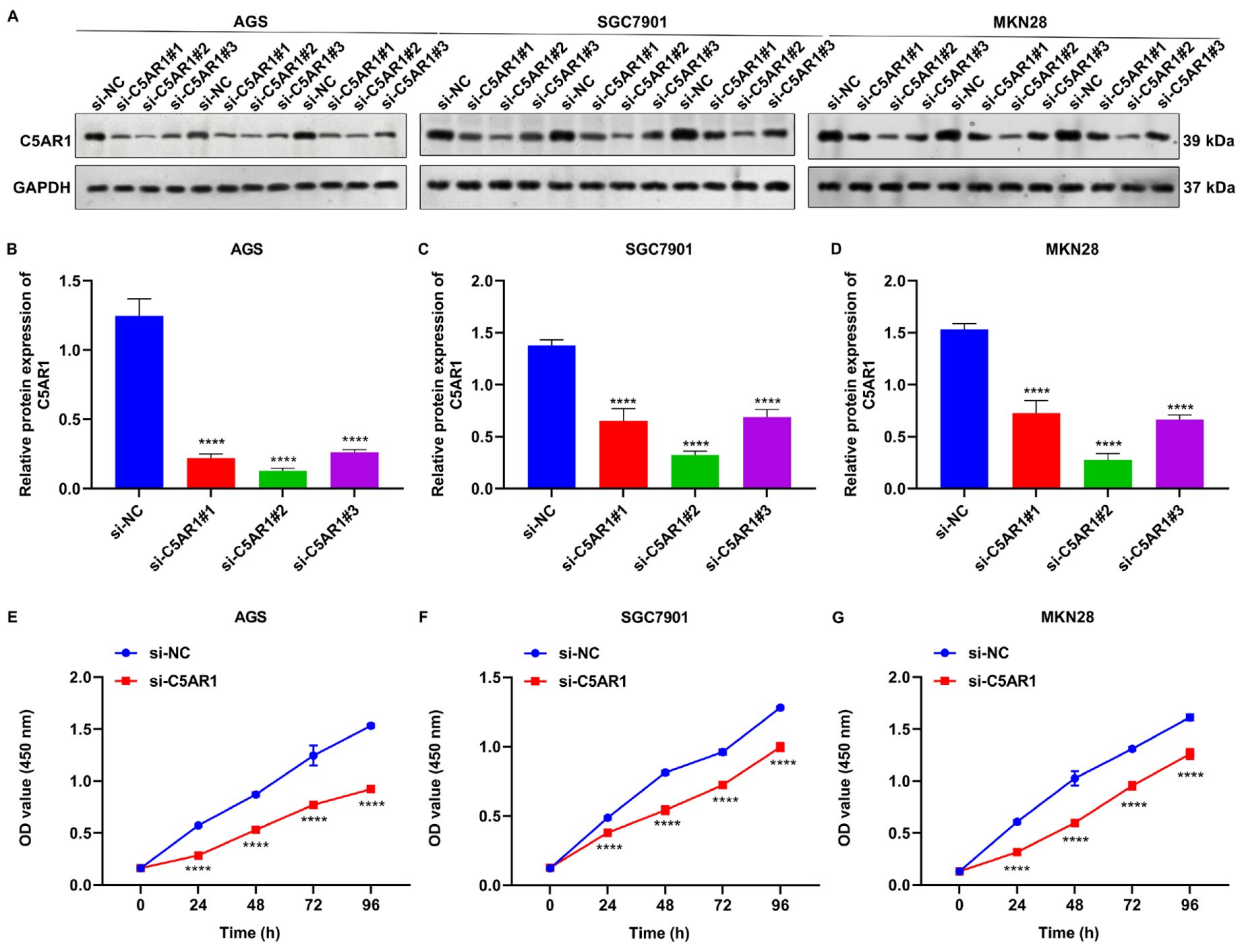
Figure 6 *C5AR1* knockdown prevents GC cell growth (A‒D) Western blot analysis of C5AR1 expressions in AGS, SGC7901 and MKN28 cells transfected with si-NC or si-C5AR1. (E‒G) CCK-8 assays for 0, 24, 48, 72 and 96 h-cell viability in AGS, SGC7901 and MKN28 cells transfected with si-NC or si-C5AR1. ****P<0.0001.

However, the biological role of C5AR1 in GC has not been determined. Thus, we next investigated the function of C5AR1 in the malignant behaviors of GC cells through experimental validation. Three siRNAs targeting *C5AR1* or its controls were transfected into AGS, SGC7901 and MKN28 cells. Consequently, si-C5AR1#2 was proven to have the optimal efficacy in knocking down *C5AR1* in three GC cell lines ( Figure 6A‒D). *C5AR1*-knockdown AGS, SGC7901 and MKN28 cells exhibited impaired proliferative capacity ( Figure 6E‒G). Thus, it was inferred that targeting *C5AR1* potentially prevents GC cell growth.

### *C5AR1* knockdown elicits intracellular ROS accumulation in GC cells

**Figure 7:**
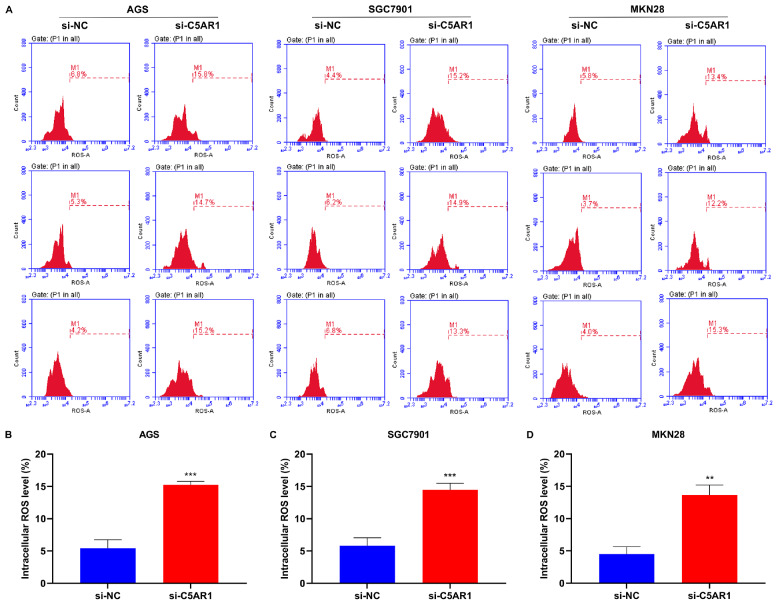
Figure 7 *C5AR1* knockdown elicits intracellular ROS accumulation (A‒D) Flow cytometry for estimation of DCFH-DA-labelled ROS levels in AGS, SGC7901 and MKN28 cells transfected with si-NC or si-C5AR1. **P<0.01 and ***P<0.001.

ROS are implicated in the formation of NETs [34]. Next, we investigated whether C5AR1 influenced intracellular ROS accumulation. Consequently, in the context of *C5AR1* knockdown, intracellular ROS levels were notably elevated in AGS, SGC7901 and MKN28 cells ( Figure 7), thus demonstrating the function of C5AR1 in modulating ROS accumulation within GC cells.

### *C5AR1* knockdown hinders the aggressiveness of GC cells

**Figure 8:**
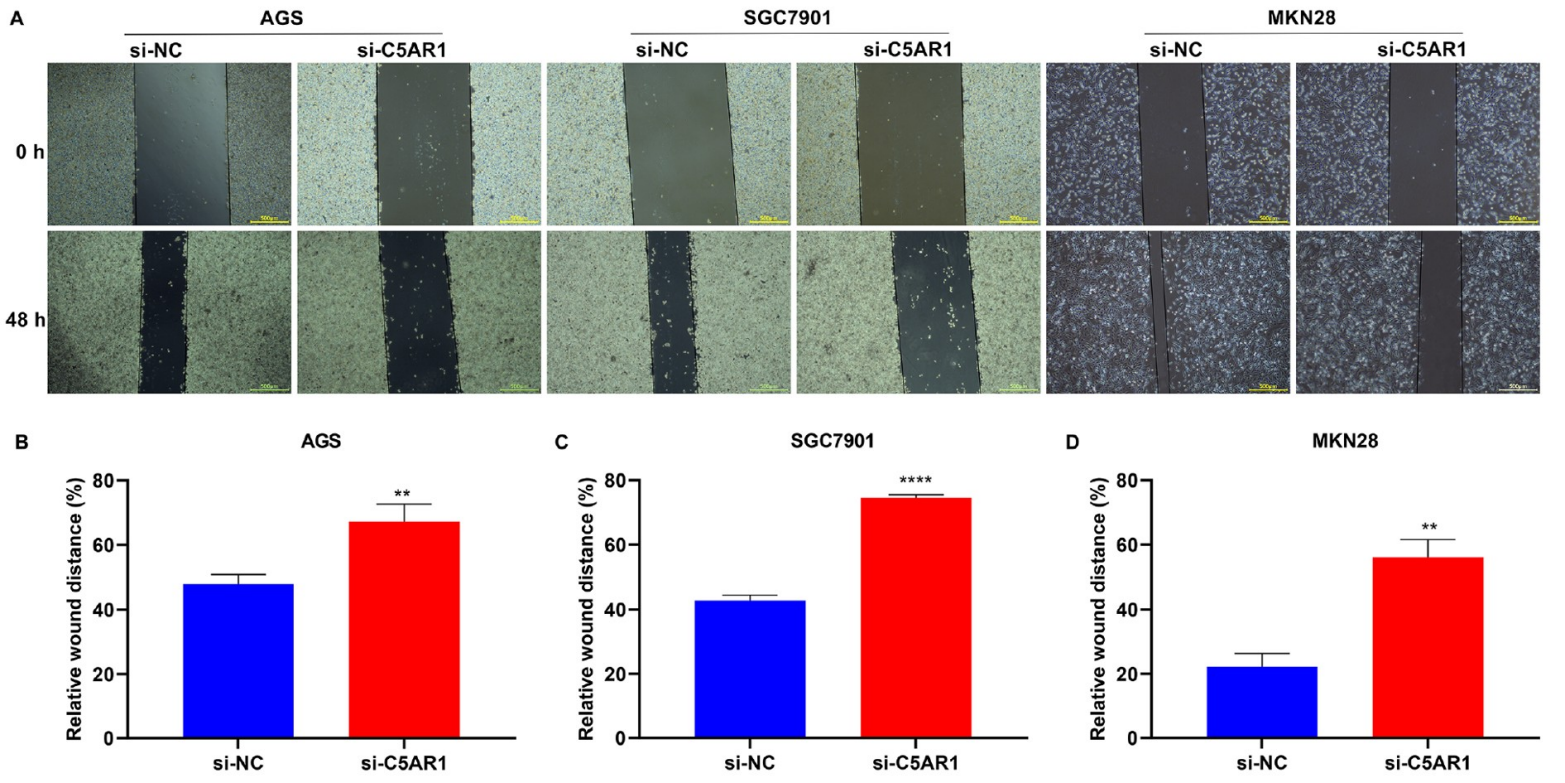
Figure 8 C5AR1 suppression impairs the aggressiveness of GC cells (A) Representative 0- and 48-h wound healing images of AGS, SGC7901 and MKN28 cells transfected with si-NC or si-C5AR1. Bar, 500 μm. (B‒D) Quantification of the relative wound healing distance in the three GC cell lines transfected with si-NC or si-C5AR1. **P<0.01 and ****P<0.0001.

Based upon the results of wound healing experiments, *C5AR1* knockdown effectively elevated the relative wound distance in AGS, SGC7901 and MKN28 cells ( Figure 8). Taken together, these findings suggested that suppression of C5AR1 expression hinders the aggressiveness of GC cells.

### *C5AR1* knockdown impairs the EMT phenotype in GC cells

**Figure 9:**
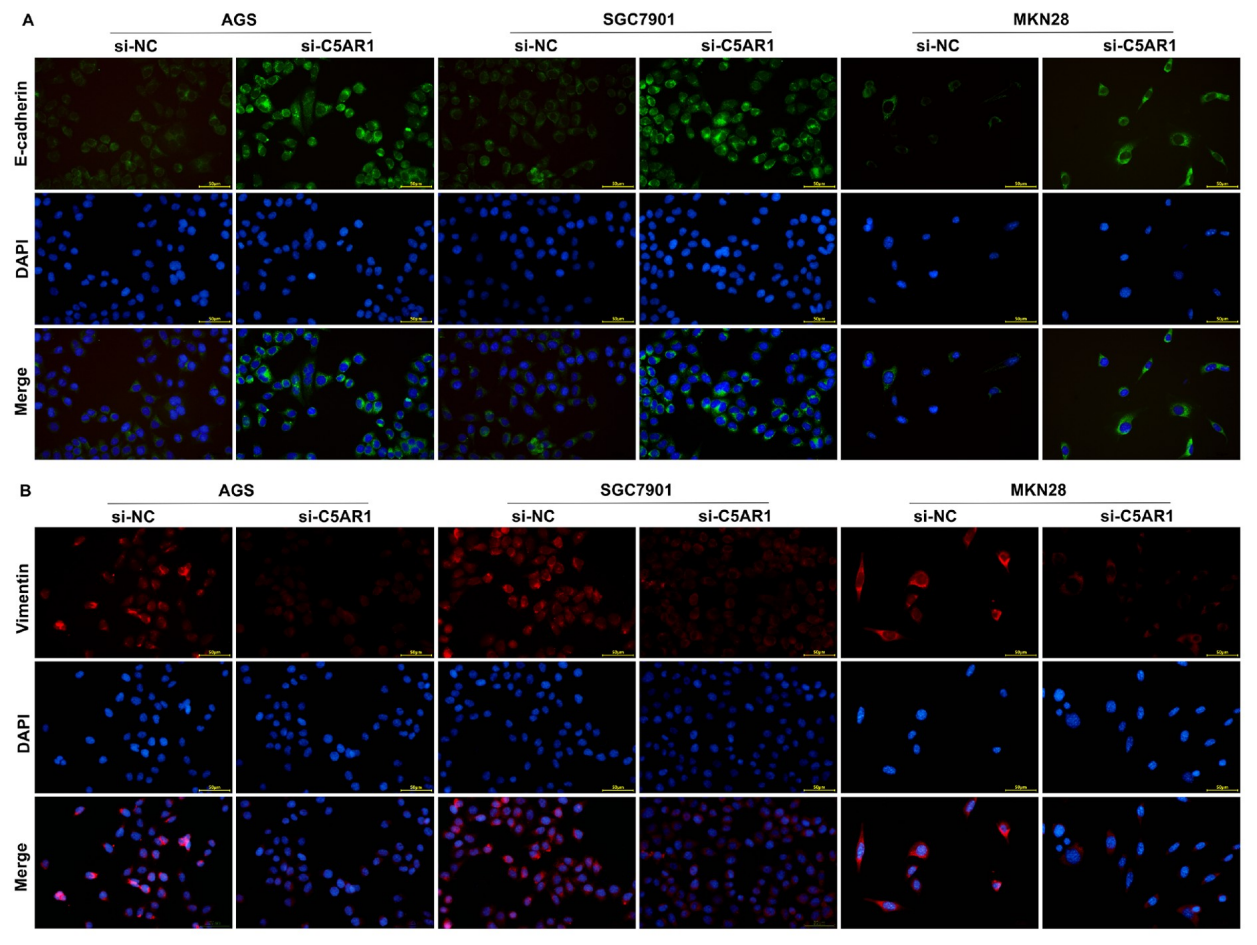
Figure 9 C5AR1 inhibition impairs the EMT phenotype in GC cells (A,B) Immunofluorescence analysis of E-cadherin and Vimentin expressions in AGS, SGC7901 and MKN28 cells transfected with si-NC or si-C5AR1. Scale bar: 50 μm.

E-cadherin, a transmembrane glycoprotein, participates in cell-cell adhesion and EMT, while Vimentin is highly expressed in mesenchymal cells and is positively associated with tumor metastasis [35]. *C5AR1* knockdown effectively elevated E-cadherin expression and attenuated Vimentin expression in AGS, SGC7901 and MKN28 cells ( Figure 9), indicating that aberrantly expressed C5AR1 might be involved in mediating the EMT phenotype of GC cells and even tumor metastasis.

## Discussion

GC has notable molecular heterogeneity, aggressive behaviors and therapeutic resistance, and thus remains a barrier to clinical management [36]. NET formation has been proven to be crucial for GC growth [16], the metastatic cascade [ 17‒ 19] and cancer-associated thrombosis [20]. Nonetheless, the heterogeneity in NETs remains unclear. Herein, this work proposed a novel NET-based molecular classification system for GC and stratified GC patients into two clusters, which might assist in choosing anti-NET therapy for GC patients.

Clinically, the two NET-based clusters exhibited heterogeneous clinical traits and prognostic outcomes. Carcinogenic signaling pathway activity was greater in cluster 1 than in cluster 2, reflecting worse survival outcomes in these patients. Somatic variants were also heterogeneous in these two clusters. In particular, mutant TP53, CDKN2A, PEG3, DMXL1, TRPV4, FMO2, and LRRC3 were more frequent in cluster 1, with more frequent mutations in SOX5, CDH1, and NCDN in cluster 2. To quantify the NETs-based classification, a logistic regression model classifier composed of C5AR1, CSF1R, CSF2RB, CYBB, HCK, ITGB2, LILRB2, MNDA, MPEG1, PLEK, SRGN, and STAB1 was eventually built, which was shown to possess possible clinical implications in NET-based subtyping, thus assisting personalized anti-NET therapy.

Currently, platinum-containing regimens are still the most common treatment for advanced GC, but chemical resistance remains one of the major reasons for treatment failure despite extensive research on the molecular mechanisms underlying chemotherapy resistance [37]. Thus, novel small-molecule agents are urgently needed for GC patients. Here, we found that cluster 2 exhibited stronger sensitivity to BI-2536, while cluster 1 was more sensitive to KU-55933, ribociclib, AZD8186, AZD8055, NU7441, SB216763, BMS-754807, AZ960, entospletinib, ZM447439, CZC24832, AMG-319, GSK269962A, WZ4003, JQ1, RVX-208, and PRT062607. Preclinical and clinical evidence has proven the therapeutic efficacy of these agents in GC. For instance, BI2536 acts as an effective and selective inhibitor of PLK1 and synergizes with cisplatin to treat GC [38]. KU-55933 suppresses ataxia telangiectasia mutated, thus preventing p53 phosphorylation [39]. A reduction in ATM kinase activity induced by KU55933 enhances olaparib sensitivity in GCs with depleted or inactivated p53 [40]. Ribociclib functions as a cyclin-dependent kinase 4/6 inhibitor for first-line therapy against HR ^+^/HER2 ^‒^ advanced breast tumors, and its bioavailability is not influenced by gastric pH alterations or food intake [41]. Based upon a phase Ib/II cohort (KCSG ST18-20), AZD8186 (a selective PI3Kβ/δ inhibitor) plus paclitaxel was well tolerated by advanced GC patients [42].

ICB therapy has resulted in a durable clinical response in a minority of GC patients [43]. The two NET-based clusters were heterogeneous in terms of the immune microenvironment, with more abundant immune and stromal components in cluster 1. The T-cell-inflamed phenotype is positively associated with the effectiveness of ICB [44]. In addition, the TIDE score is negatively linked to the ICB response. In accordance with the lower T-cell-inflamed score and lower TIDE score, cluster 2 patients were inferred to respond better to ICB but more likely to experience therapeutic resistance. Hence, more reliable biomarkers of ICB are urgently needed.

The feature genes exhibited strong interactions and heterogeneous expression between NET-based clusters in GC tumors. Among them, C5AR1, CSF1R, CSF2RB, CYBB, HCK, ITGB2, LILRB2, MNDA, MPEG1, PLEK, and SRGN were proven to be prominently upregulated in GC cells, with notable downregulation of STAB1 level. Several feature genes have been experimentally verified in prior studies. For instance, CSF1R participates in modulating cancer stemness, the immunosuppressive microenvironment and metastasis in GC [45]. A genetic reduction in the SRC kinase HCK prevents STAT3-based GC growth [46]. Blockade of the complement receptor C5AR1 enables tumor-associated macrophages to be reprogrammed and reinvigorated CD8 ^+^ T-cell-mediated antitumor immunity, thereby synergizing with anti-PD-1 therapy for GC tumor eradication [47]. After further verification of C5AR1, we found that specific suppression of C5AR1 prevented GC growth and provoked intracellular ROS generation. Numerous studies have explored the molecular mechanisms governing the process of GC invasion and metastasis; for example, CORO1C enables the recruitment of phospho-PAK4 at serine 99 to the leading edge and promotes the migration of GC cells [48]. This study revealed that C5AR1 inhibition impaired the aggressiveness and EMT phenotype of GC cells. Taken together, these findings indicate that C5AR1 functions as a treatment target for GC. Notably, C5AR1 showed higher expression in NET-based cluster 1 than in cluster 2. Thus, therapeutically targeting C5AR1 might be effective for GC patients in cluster 1. Our findings could assist in selecting appropriate treatment targets and regimens for GC.

Nevertheless, several limitations need to be acknowledged in this study. Despite the external verification, additional GC samples are needed to prove the reliability of the NET-based classification. Moreover, the biological roles of C5AR1 and other feature genes need to be investigated in the future.

In summary, the results of this study revealed that NET formation is heterogeneous in GC, and a novel NET-based classification was proposed, which can be estimated by a machine learning-based classifier. Additionally, C5AR1 was proven to mediate GC growth and metastatic spread. Despite this, our conclusions warrant further in-depth investigations.

## Supporting information

23482Supplementary_Table_S1

23482-z_Supplementary_Data

## Supplementary Data

Supplementary data is available at *Acta Biochimica et Biophysica Sinica* online.
