# Supplementary material for: Identification of neutrophil extracellular trap-driven gastric cancer heterogeneity and C5AR1 as a therapeutic target: Identification of NET-driven GC heterogeneity and C5AR1 as a therapeutic target
Source: Acta Biochim Biophys Sin (Shanghai). 2024 Mar 1;56(4):538–50. doi: 10.3724/abbs.2023290 (PMC11090850; doi:10.3724/abbs.2023290)
Supplement: 23482-z_Supplementary_Data [file 23482-z_Supplementary_Data.pdf]

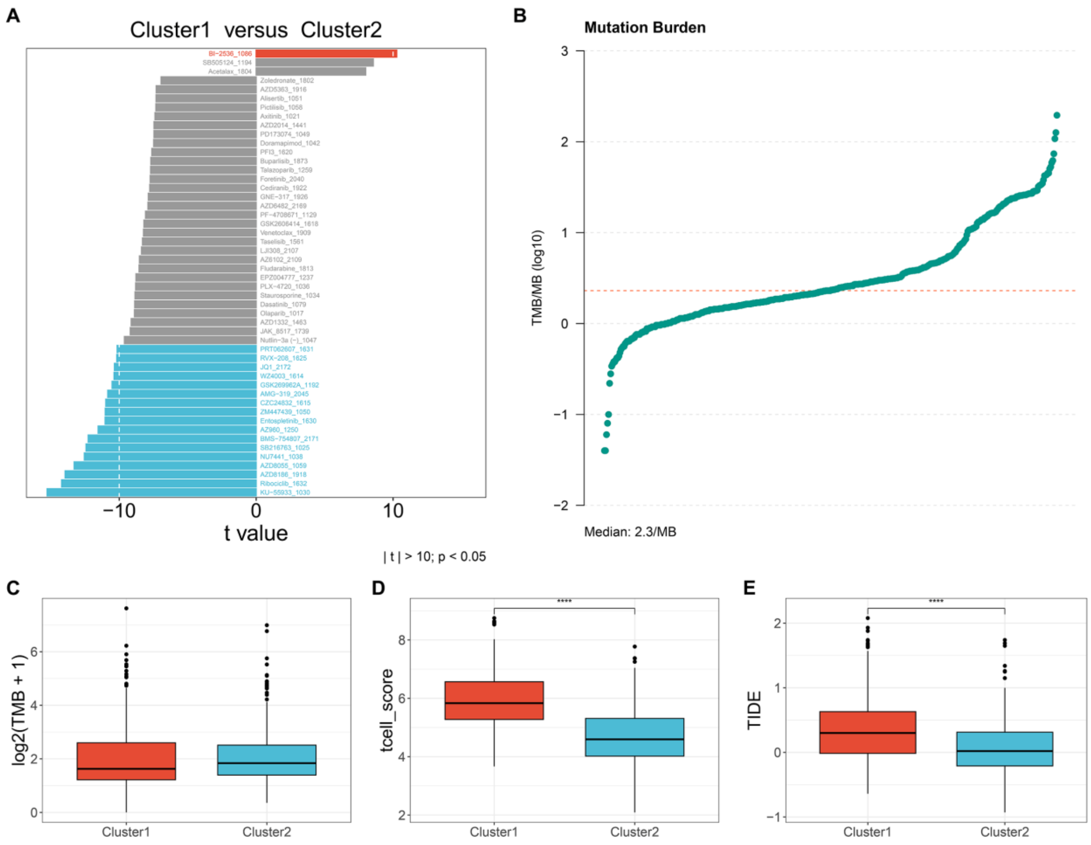

Supplementary Figure S1. Heterogeneous therapeutic responses in the two NET-based clusters (A) Differential IC_{50} values of small-molecule agents between clusters. The threshold was set as |t|>10 and *P*<0.05. (B) Distribution of TMB across TCGA-STAD tumor types. The median TMB was 2.3/MB. (C) Comparison of TMB between the two clusters. (D,E) Differential T-cell-inflamed and TIDE scores between clusters. ****P<0.0001.

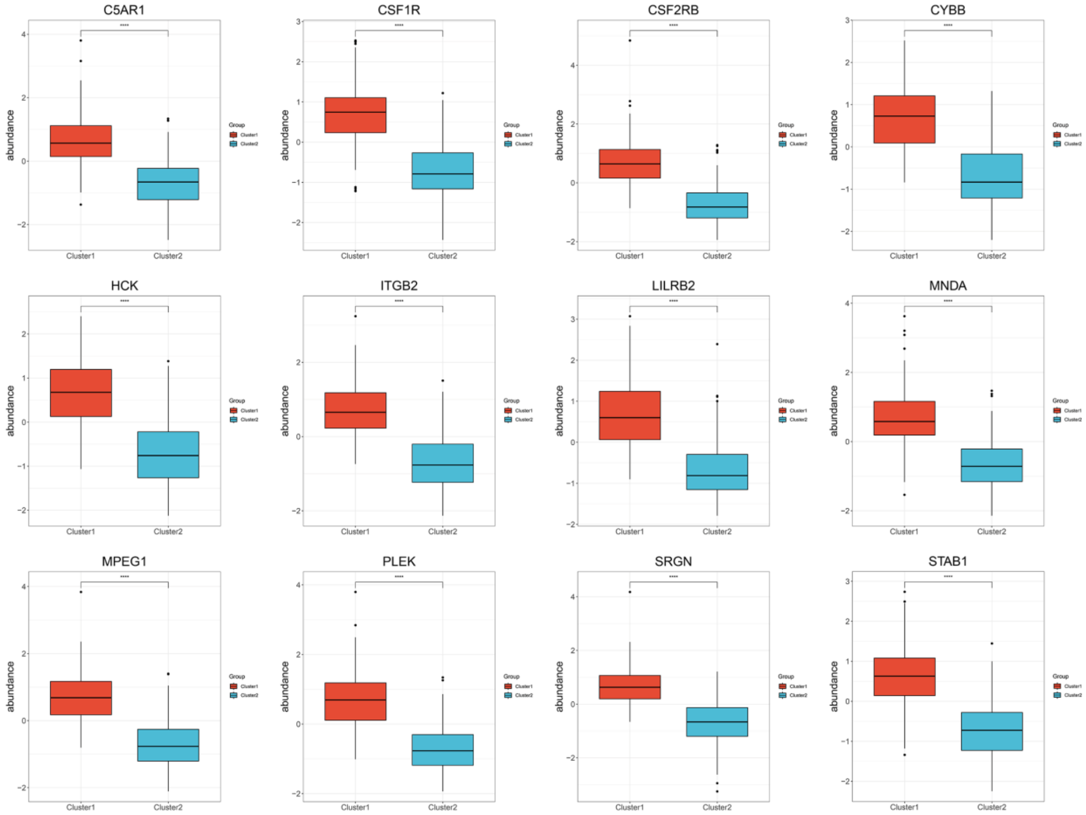

Supplementary Figure S2. Heterogeneous expressions of C5AR1, CSF1R, CSF2RB, CYBB, HCK, ITGB2, LILRB2, MNDA, MPEG1, PLEK, and SRGN in two NET-based clusters ****P<0.0001.

Supplementary Table S1. Information on 35 NET-associated genes
